# Porous GaN: Anion-Specific Electrochemical Etching Mechanisms and Morphological Control

**DOI:** 10.1021/acsami.5c18520

**Published:** 2025-11-13

**Authors:** Thom R. Harris-Lee, Ben Thornley, Jiawei Zhang, Menno J. Kappers, Rachel A. Oliver

**Affiliations:** † Department of Materials Science and Metallurgy, 2152 University of Cambridge, Cambridge, CB3 0FS, U.K.

**Keywords:** electrochemical etching, GaN, n-type, porous, anion, dissociation, electrolyte

## Abstract

Porous GaN has emerged as a promising material for enhancing the performance of optoelectronic devices and broadening the range of possible GaN applications. However, the electrochemical etching (ECE) process used to create porosity remains poorly understood, particularly regarding the impact of the chemical environment on pore morphology. Here, the controlled ECE of n-type GaN is systematically investigated across a range of etchant chemicals and pH values. It is shown that the identity, speciation, and relative concentrations of anionic species play dominant roles in dictating porous morphology. Through deliberate manipulation of anion compositions within an etchant solution, for example, by adjusting initial polyprotic acid concentration and/or addition of a conjugate salt, porous morphology and surface structure can be controlled and tuned effectively. Further, ECE-generated current oscillations, previously interpreted as evidence for an oxidation–dissolution ECE mechanism, are shown to correlate with the presence of dynamic anion equilibria, providing an additional mechanistic interpretation of n-type GaN ECE. This furthered understanding enables more tailored and application-specific control over porous structure, offering opportunities for optimized, bespoke GaN-based porous architectures.

## Graphical abstract

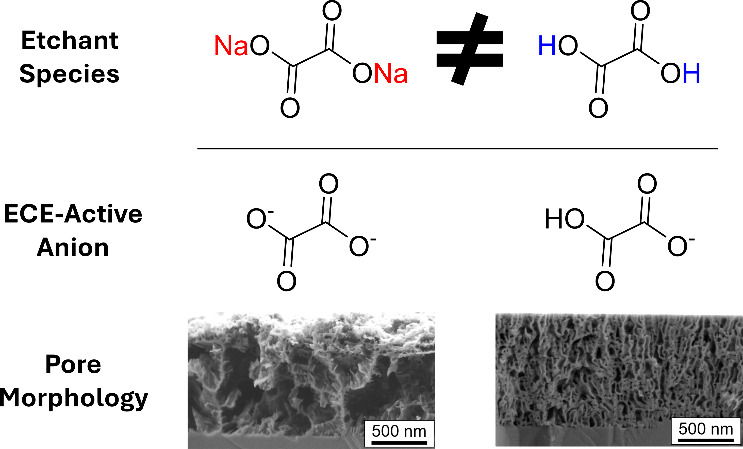

## 1 Introduction

Gallium nitride (GaN) is a wide-bandgap (3.4 eV) material known for its high thermal stability, chemical resistance, and excellent electronic and optoelectronic properties, being widely used in efficient LED lighting and high-power transistors.^1–3^ Porosification of GaN can aid in overcoming some common issues such as difficult strain management,^4,5^ as well as broadening the range of potential applications to include composite formation via infiltration,^6,7^ catalysis,^8,9^ and sensing.^10,11^ n-Type GaN thin films can be made porous by a conductivity-selective electrochemical etching (ECE) process (eq 1).

1 $$2{\mathrm{GaN}}_{\left(s\right)}\to 2{{\mathrm{Ga}}^{3+}}_{\left(\mathrm{aq}\right)}+N_{2\left(g\right)}+6e^{-}$$

Porosity can be tuned by a range of ECE conditions, including applied potential, etchant solution species, etchant solution concentration, and n-type GaN free carrier density.^12^ However, due to limited understanding of the underlying ECE mechanism, porosity control and optimization currently rely on empirical observations, making it very difficult to develop bespoke porous morphologies tailored to a specific application. It is noted that ECE of p-type GaN is significantly more difficult than n-type, evident from the very limited published reports showing success,^13,14^ and will therefore not be considered herein.

The present ECE mechanistic understanding is widely based on an oxidation–dissolution ‘current-burst model’, which predicts that an electrochemical reaction self-oxidizes conductive n-type GaN to insulating gallium oxide (Ga_2_O_3_), which is then chemically dissolved by the etchant solution.^15–18^ This mechanism has been inferred as true for GaN based on the same mechanism being concluded for the ECE of silicon in a HF etchant solution.^17,19^ However, the only evidence for this same mechanism in n-type GaN is an oscillating etching current often produced as a result of the ECE process.^12,16,17^ It is proposed that the current increases while GaN is being oxidized to Ga_2_O_3_ as a result of the electrochemical reaction, but once the thickness of the insulating Ga_2_O_3_ formed reaches a critical threshold, the current will drop as the electrochemical reaction is inhibited.^12,19^ Ga_2_O_3_ is then chemically dissolved by the etchant solution, revealing new n-type GaN for the electrochemical reaction to resume and hence increase the measured current. This mechanism is unproven for n-type GaN ECE, and alternative mechanisms have been proposed.^20,21^

It is known that different etchant chemicals will influence the ECE mechanism to result in different porous morphologies, with a range of etchant chemicals having been used including H_2_C_2_O_4_,^21–23^ NaCl,^24^ HNO_3_,^22,24^ KOH,^23^ HF,^16,25^ and H_2_SO_4_.^20,26^ The influence of etchant species has been previously reported, suggesting that the anion species from the electrolyte, not the cation species^24^ nor solution pH,^27^ controls the porous structure. While these studies do show a changing pore morphology based on the etchant species used, they compare a narrow range of chemicals and do not control all other variables. Herein, by systematically studying a comprehensive series of acidic, alkaline, and neutral electrolytes, it is demonstrated that the observed differences in porous structures correspond directly to the dominant anionic species in solution, which in acids is governed by their dissociation constants.

## 2 Experimental Section

### GaN Sample Preparation

Ga-polar GaN epilayer growth was performed by metal–organic vapor phase epitaxy (MOVPE) in an Aixtron close-coupled showerhead reactor on 2 in. (0001) sapphire substrates. The sample structure consisted of a 2.4 μm-thick layer of nominally undoped GaN, followed by a 1 μm-thick layer of highly n-type GaN with a Si concentration ([Si]) of 1.5 × 10^19^ cm^–3^. The threading dislocation density was measured at ca. 9 × 10^8^ cm^–2^ using the method described by Oliver et al.^28^

### Chemical Reagents and Electrode Materials

Oxalic acid (H_2_C_2_O_4_, Sigma-Aldrich, 99.97%), sodium oxalate (Na_2_C_2_O_4_, Thermo Fisher Scientific, 99%), sulfuric acid solution (H_2_SO_4_, Thermo Fisher Scientific, 0.25 M NIST standard), sodium sulfate (Na_2_SO_4_, Sigma-Aldrich, >99%), phosphoric acid (H_3_PO_4_, Sigma-Aldrich, 99.99%), sodium phosphate tribasic (Na_3_PO_4_, Sigma-Aldrich, 96%), nitric acid solution (HNO_3_, Honeywell-Fluka, 70% ACS grade), potassium nitrate (KNO_3_, Thermo Fisher Scientific, >99%), hydrochloric acid solution (HCl, Sigma-Aldrich, 37% ACS grade), sodium chloride (NaCl, Thermo Fisher Scientific, >99%), acetic acid (CH_3_COOH, Sigma-Aldrich, >99.7%), sodium acetate (CH_3_COONa, Sigma-Aldrich, >99%), sodium hydroxide (NaOH, Thermo Fisher Scientific, >98%), and potassium hydroxide (KOH, VWR Chemicals, 88%) were used as supplied by the manufacturer. All aqueous solutions were prepared with ultrapure deionized (DI) water (resistivity = 22.2 MΩ cm at 25 °C, Direct-Q 3 UV water purification system, Milli-Q).

A 10 × 10 × 0.1 mm platinum plate (Ossila, 99.99%) was used as the counter electrode (CE). A 3 M Ag/AgCl electrode (Ossila) was used as the reference electrode (RE). Note that, unless otherwise stated, all potentials herein are referenced to the 3 M Ag/AgCl RE scale. Indium wire (In, Thermo Fisher Scientific, >99.998%) was melted and soldered to the n-type GaN surface to form the conductive contact.

### Electrochemical Etching

ECE was carried out on a Gamry Reference 3000 potentiostat connected to a three-electrode electrochemical cell containing a Pt plate CE and 3 M Ag/AgCl RE (Figure 1). Electrical connection was formed on an ∼0.6 × 1.2 cm^2^ GaN sample by soldering an indium metal contact on one end, which was then connected into the electrochemical cell as the working electrode (WE) and submerged into electrolyte approximately halfway. An anodic bias of 8 V vs Ag/AgCl and 50 mL of fresh 100 mmol dm^–3^ (mM) solutions were used for every experiment, unless stated otherwise. During ECE, etchant solutions were stirred at 300 rpm using a magnetic stirrer bar. After ECE, samples were washed thoroughly with DI water, followed by drying under N_2_.

**Figure 1:**
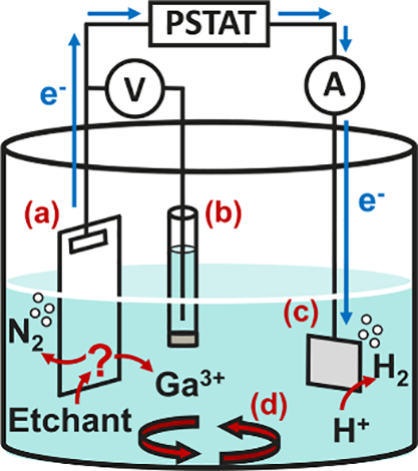
Schematic of the electrochemical etching cell. (a) n-Type GaN WE with In contact, (b) 3 M Ag/AgCl RE, (c) Pt plate CE, and (d) 300 rpm stirred etchant solution.

Chronoamperometry curves display the current generated during the ECE at a constant applied voltage as a function of time. Herein, for accurate comparison of etching current magnitudes between samples, all etching currents have been normalized to the original planar geometric surface area of the etched sample region (*I*_GA_). Note that, due to the ongoing porosification process, the sample surface area is continuously increasing; hence, the measured current cannot be accurately converted to current density. Geometric area-normalized charge (*Q*_GA_) was calculated by integrating the *I*_GA_ traces, representing the total charge passed throughout the ECE process, and therefore the total amount of GaN etched.

### Physical Characterization

Cross-sectional and surface porous morphologies were obtained by scanning electron microscopy (SEM, Zeiss Gemini 300) using secondary electron (SE) imaging. Unless otherwise stated, all plane-view surface images were taken at a 45° sample tilt. High-resolution surface images were generated by atomic force microscopy (AFM, Bruker Dimension Icon) in PeakForce tapping mode by using ScanAsyst HPI tips from Bruker.

## 3 Results and Discussion

### 3.1 Etching in Oxalic Acid vs Sodium Oxalate

ECE of n-type GaN is often conducted using oxalic acid (H_2_C_2_O_4_) as the etchant solution, with previous reports highlighting the broad range of accessible porous morphologies, simply by adjusting the applied voltage and/or Si doping concentration.^12,24^ While the mechanism of n-type GaN ECE is not fully understood, it is known that different etchant species (e.g., H_2_C_2_O_4_, HNO_3_, NaOH) yield distinct etching behaviors and pore structures.^24,27^ To investigate the role of etchant chemical structure, ECE was conducted on 1 μm-thick n-type GaN ([Si] = 1.5 × 10^19^ cm^–3^) at 8 V vs a Ag/AgCl reference electrode, using 100 mM H_2_C_2_O_4_ and 100 mM sodium oxalate (Na_2_C_2_O_4_). Despite the structural similarity of H_2_C_2_O_4_ and Na_2_C_2_O_4_, both of which are based on the C_2_O_4_^2–^ anion, the chronoamperometry traces (Figure 2a) and resulting porous morphologies (Figure 2b,c) differ substantially.

**Figure 2:**
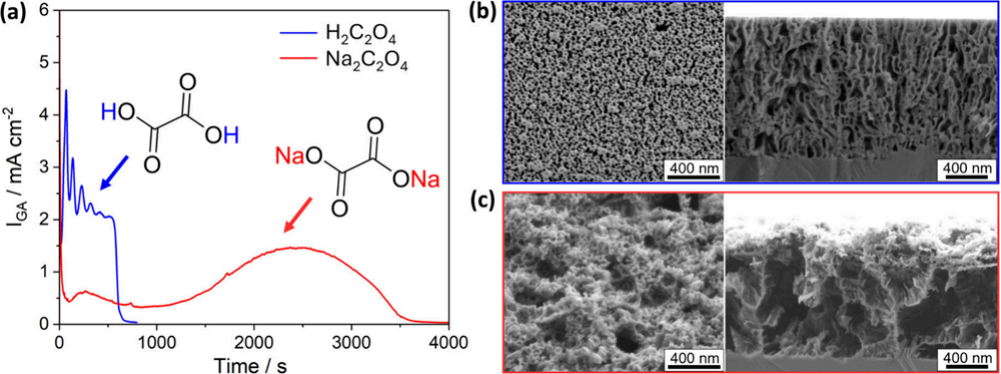
(a) Chronoamperometry data for ECE of 1 μm n-type GaN ([Si] = 1.5 × 10^19^ cm^–3^) etched at 8 V vs Ag/AgCl in (blue) 100 mM H_2_C_2_O_4_ and (red) 100 mM Na_2_C_2_O_4_. SEM plan view and cross-section images for the resulting porous GaN from (b) 100 mM H_2_C_2_O_4_ and (c) 100 mM Na_2_C_2_O_4_.

While the surface etched by H_2_C_2_O_4_ contains pores, it remains relatively flat with a consistent pore distribution (Figure 2b). Na_2_C_2_O_4_, however, produces more aggressively etched surfaces, with less well-defined pathways and more irregular topography (Figure 2c). Large pores within the overall porous surface structure form in the sample etched by Na_2_C_2_O_4_, with diameters of ∼320 nm, compared to an ∼30 nm average diameter when using H_2_C_2_O_4_. These differences are also evident in the cross-sectional images, where H_2_C_2_O_4_ yields a relatively consistent, overall vertically oriented porous morphology, while Na_2_C_2_O_4_ produces significantly larger pore volumes with little directional preference, leaving behind only thin, branching pillars of unetched GaN.

H_2_C_2_O_4_ completes etching after ∼750 s and exhibits *I*_GA_ oscillations (Figure 2a), which have previously been associated with the current-burst model. In contrast, Na_2_C_2_O_4_ requires ∼3600 s to complete etching and no *I*_GA_ oscillations are observable. Instead, the *I*_GA_ trace contains two distinct peaks (at ∼250 and ∼2450 s). Selective ECE, manually stopped at the end of the first *I*_GA_ peak, reveals that this feature corresponds to ECE of only the upper ∼250 nm of the n-type GaN layer, followed by the second larger peak from ECE of the remaining 750 nm ‘bulk’ (SI, Figure S1). The origin of this difference between the ‘surface’ and ‘bulk’ regions is not yet understood and will be the subject of further study.

The considerable difference in porous morphologies between H_2_C_2_O_4_ and Na_2_C_2_O_4_ etchants provides insight into the underlying ECE mechanism. Three major variables differ between the two solutions which could affect ECE: (i) the etchant solution pH (100 mM H_2_C_2_O_4_: pH 1.29, 100 mM Na_2_C_2_O_4_: pH 8.40); (ii) the anionic (negative) species present in solution following dissociation in DI water (H_2_C_2_O_4_ dissociates as a weak acid to both HC_2_O_4_^–^ and C_2_O_4_^2–^, while Na_2_C_2_O_4_ fully dissociates to yield only C_2_O_4_^2–^); (iii) the identity of the cationic (positive) species (H^+^ for H_2_C_2_O_4_, Na^+^ for Na_2_C_2_O_4_). Note that the pH of Na_2_C_2_O_4_ is not neutral despite dissociating fully to C_2_O_4_^2–^, as this anion will undergo a further hydrolysis reaction in water to form OH^–^ and HC_2_O_4_^–^. However, the concentrations of OH^–^ and HC_2_O_4_^–^ produced are negligible and will not influence the ECE mechanism (e.g., only 2.54 μM of each are produced in a 100 mM Na_2_C_2_O_4_ solution).

Differences in the cationic species are unlikely to directly influence the ECE mechanism. Since ECE occurs under anodic bias, positively charged cations would need to overcome electrostatic repulsion from the GaN surface, making their direct participation in the reaction unfavorable. Additionally, the formation of intermediate complexes involving electrolyte cations is unlikely due to the already high positive charge density of Ga^3+^, which is likely also involved within any intermediate as part of the ECE mechanism.^29^

To confirm this, ECE was performed using NaCl and KCl, as well as NaOH and KOH etchants (Figure 3). NaCl and KCl etchants produced very similar surface and bulk morphologies, exhibiting somewhat aligned rows of pores with small diameters (∼10 nm), inferred to be extending into the plane of the film (Figure 3a,b i,ii). The AFM images (Figure 3a,b iii) show surfaces that appear unetched (RMS roughness values of ca. 0.34 nm and ca. 0.33 nm for NaCl and KCl, respectively), indicating that ECE is occurring via threading dislocations only, followed by lateral etching outward from the dislocation cores.^30^ Differences seen in the cross-sectional images, particularly at low magnification, largely reflect how the cleaving process (where etched samples are fractured and the fracture surface is examined) has proceeded, not the porous structure itself. Further, comparison of Figure 3a with Figure 3c reveals entirely different pore morphologies, despite NaCl and NaOH possessing the same cation (Na^+^), therefore reinforcing the negligible impact of the cation compared to anion species.

**Figure 3:**
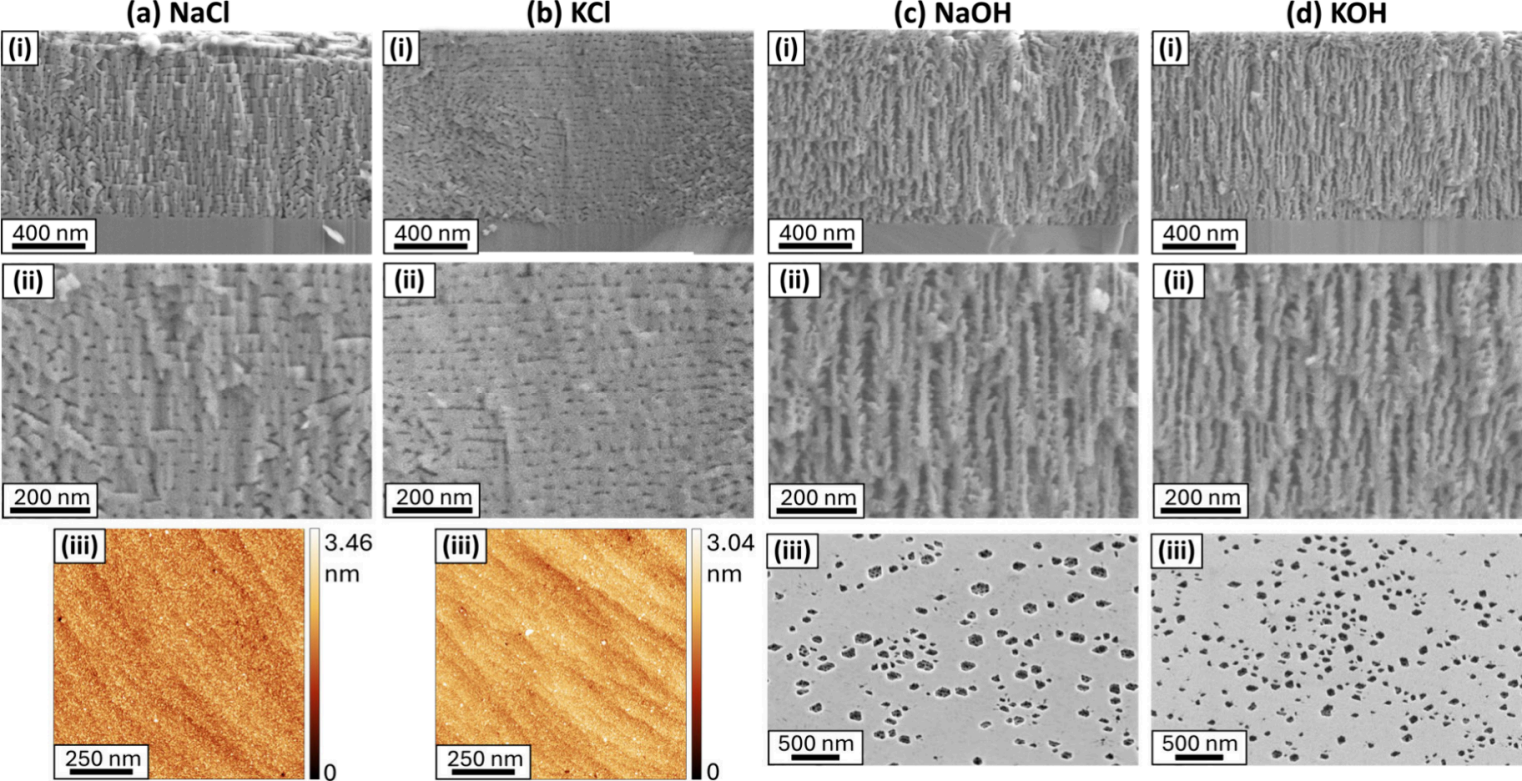
SEM cross section (a–d i, ii) and surface AFM (a,b iii) and surface SEM (c,d iii) images for 1 μm n-type GaN ([Si] = 1.5 × 10^19^ cm^–3^) etched at 8 V vs Ag/AgCl in 100 mM (a) NaCl, (b) KCl, (c) NaOH, and (d) KOH.

ECE with NaOH produced slightly larger surface pits than KOH (∼230 nm vs ∼130 nm diameter, respectively) and marginally larger bulk pore diameters (Figure 3c,d). However, despite these differences, the overall porous morphology remained very similar, with vertically oriented pores and little branching. This suggests that, while the cation species may slightly influence local pore features, it does not significantly alter the underlying ECE mechanism and hence overall pore structure and can therefore not explain the difference between etching with H_2_C_2_O_4_ and Na_2_C_2_O_4_. To investigate the role of the H^+^ cation, particularly in comparison to Na^+^ as in H_2_C_2_O_4_ and Na_2_C_2_O_4_, the influence of the solution pH (i.e., the concentration of H^+^) must also be considered.

### 3.2 pH-Controlled Etching

In a conventional ECE setup using a single etchant, the pH of the solution is not controlled.^12^ To investigate whether solution pH has an impact on ECE, a pH buffer solution based on the oxalate anion can be used as the etchant, offering enhanced pH control, particularly in highly porous regions, where electrolyte diffusion is restricted. Buffer solutions mitigate local pH fluctuations caused by H^+^ production/consumption in the ECE process by utilizing the dynamic equilibrium of weak acid dissociation.^31^ A range of oxalate buffer solutions with different controlled pH levels were made by mixing H_2_C_2_O_4_ and its conjugate base, Na_2_C_2_O_4_, in different ratios. Importantly, a total buffer solution concentration of 100 mM was consistently achieved since the molar ratio of H_2_C_2_O_4_ to Na_2_C_2_O_4_, not the absolute concentrations, determines the set pH. Buffer solutions with pH values ranging from pH 1 to pH 2 (see Table 1) were used as etchant solutions for ECE of 1 μm n-type GaN ([Si] = 1.5 × 10^19^ cm^–3^), etched at 8 V vs Ag/AgCl.

**Table 1:** Concentrations of H_2_C_2_O_4_ and Na_2_C_2_O_4_ Used to Make a 100 mM Oxalate-Based pH Buffer Solution at Different Controlled pH Levels and the Relative Ratio of [H_2_C_2_O_4_]/[Na_2_C_2_O_4_] for Each

pH	[H_2_C_2_O_4_]/mM	[Na_2_C_2_O_4_]/mM	Relative Ratio
1	65	35	1.85
1.29	49	51	0.95
1.5	37	63	0.59
1.75	25	75	0.33
2	16	84	0.19

Figure 4a shows *I*_GA_ traces for the ECE conducted in buffer solutions across a range of acidic pH values. As pH increases from 1 to 2, the *I*_GA_ traces transition from those resembling H_2_C_2_O_4_ etching (characterized by distinct oscillations and a duration < 1000 s) to those resembling Na_2_C_2_O_4_ etching (characterized by two broad peaks and durations > 1000 s). Interestingly, pH 1.29, 1.5, and 1.75 all resulted in similar final *Q*_GA_ values (∼1.86–1.97 mC cm^–2^), although the final *Q*_GA_ increased slightly with each higher pH value, whereas pH 1 yielded a significantly lower value (∼1.64 mC cm^–2^) and pH 2 was significantly higher (∼2.51 mC cm^–2^). The chronoamperometry trace for pH 1.5 (Figure 4) contains weaker current oscillations and an increased duration compared to lower pH solutions, suggesting a possible intermediate state containing features of both the H_2_C_2_O_4_ and Na_2_C_2_O_4_ ECE regimes. Corresponding AFM and SEM images (Figure 4b–d) reveal a morphological transition with increasing pH, from the relatively flat surface resembling ECE in H_2_C_2_O_4_ (Figure S2) at pH 1 (Figure 4b) to the substantially rougher surface (RMS roughness values for pH 1, 1.29, and 2 are ca. 8.5 nm, ca. 9.6 nm, and ca. 24.0 nm, respectively) typical of ECE in Na_2_C_2_O_2_ at pH 2 (Figure 4d). However, this transition may be correlated not only with etchant pH but also with the changing composition of buffer solution. As the pH of the buffer solution increases, the relative proportion of Na_2_C_2_O_4_ also increases, from a majority of H_2_C_2_O_2_ at pH 1 (Figure 4b), to approximately equal components at pH 1.29 (Figure 4c), to a majority of Na_2_C_2_O_4_ at pH 2 (Figure 4d).

**Figure 4:**
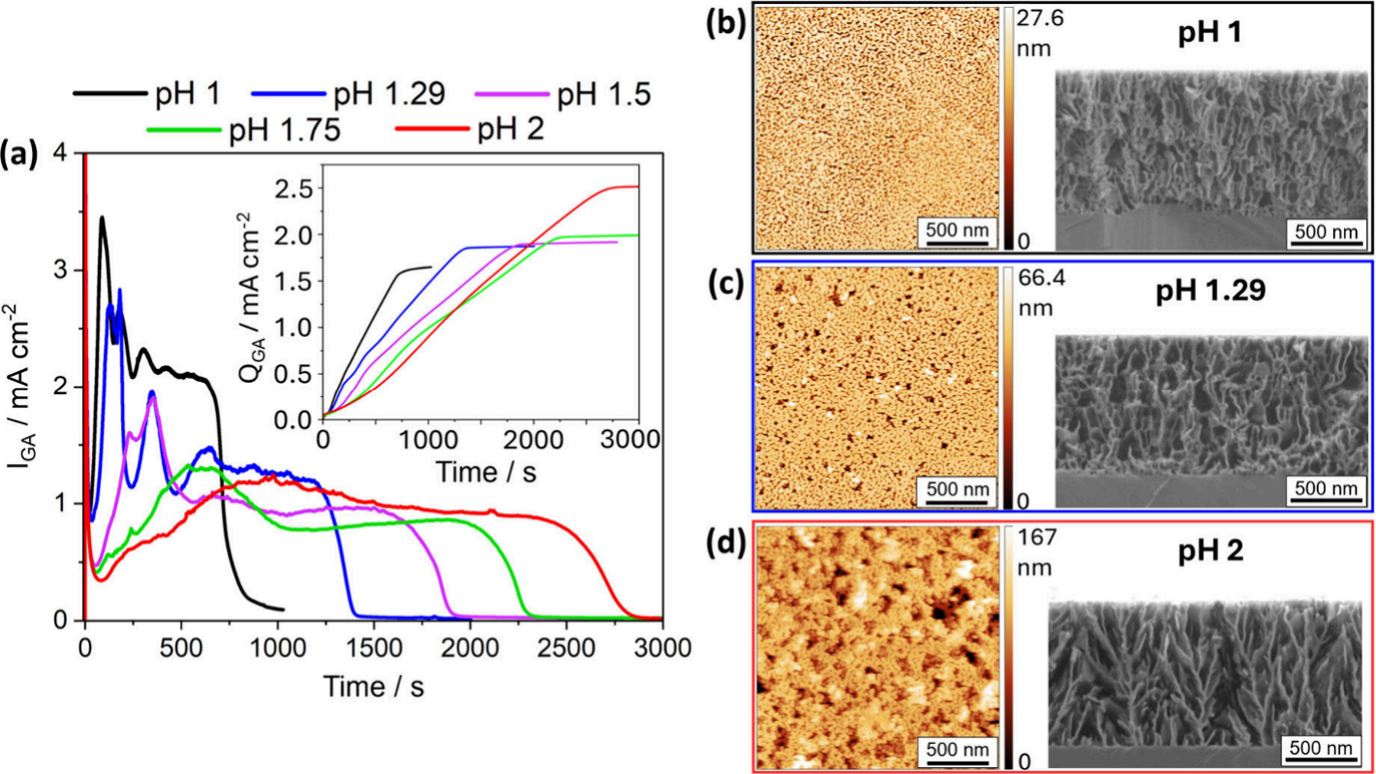
(a) Chronoamperometry data for ECE of 1 μm n-type GaN ([Si] = 1.5 × 10^19^ cm^–3^) etched at 8 V vs Ag/AgCl in 100 mM oxalate pH buffer solution set to controlled pH values of (black) pH 1, (blue) pH 1.29, (pink) pH 1.5, (green) 1.75, (red) 2; inset: charge (integrated current) traces. Surface (AFM) and cross-section (SEM) images for the resulting porous GaN from oxalate buffer solutions with pH values of (b) pH 1, (c) pH 1.29, and (d) pH 2.

To determine whether the morphological changes arise from etchant pH or from the etchant composition, additional ECE experiments were performed in solutions of H_2_C_2_O_4_ only, with decreasing concentrations (Figure 5). While the pH is no longer buffered in these solutions, decreasing the concentration of H_2_C_2_O_4_ results in a higher starting solution pH value. These experiments were conducted in a two-electrode setup at 8 V vs CE. At concentrations of 250 mM (pH 1, Figure 5a), 50 mM (pH 1.5, Figure 5b), and 20 mM (pH 1.8, Figure 5c), the resulting surface morphology appears identical, contrasting with the buffer solution results at similar pH values (Figure 4b–d, Figure S3). Even at 5 mM (pH 2.3, Figure 5d), the surface remains similar to that obtained at pH 1, in stark contrast to the highly roughened morphology resulting from ECE in the pH 2 buffer solution (RMS roughness values for 250 mM, 50 mM, 20 mM, and 5 mM are ca. 3.4, 3.0, 2.6, and 2.4 nm, respectively). Noticeable, but still minor, roughening of the surface structure only starts to occur at a concentration of 1 mM (pH 3.0, RMS roughness value of ca. 5.6 nm). At 0.5 mM (pH 3.3), the surface becomes significantly more rough, resembling the surface from ECE in pH 2 oxalate buffer. The fast transition between no change and significant surface roughening suggests that there is a critical H_2_C_2_O_4_ concentration where the etching mechanism changes to form GaN surface morphologies representing an H_2_C_2_O_4_ or Na_2_C_2_O_4_-driven ECE (Figure 2). Unlike the surface morphologies, the subsurface porous morphologies (Figure S4) begin to change significantly at 5 mM H_2_C_2_O_4_ etchant, transitioning from a highly branched morphology to a more vertically oriented structure. Again, after this transition, the pores become wider and show a greater resemblance to the morphology produced by the Na_2_C_2_O_4_ etchant than H_2_C_2_O_4_. The porous morphology changes completely when using <0.5 mM H_2_C_2_O_4_, likely due to entering an etchant anion-limited ECE mechanistic regime.

**Figure 5:**
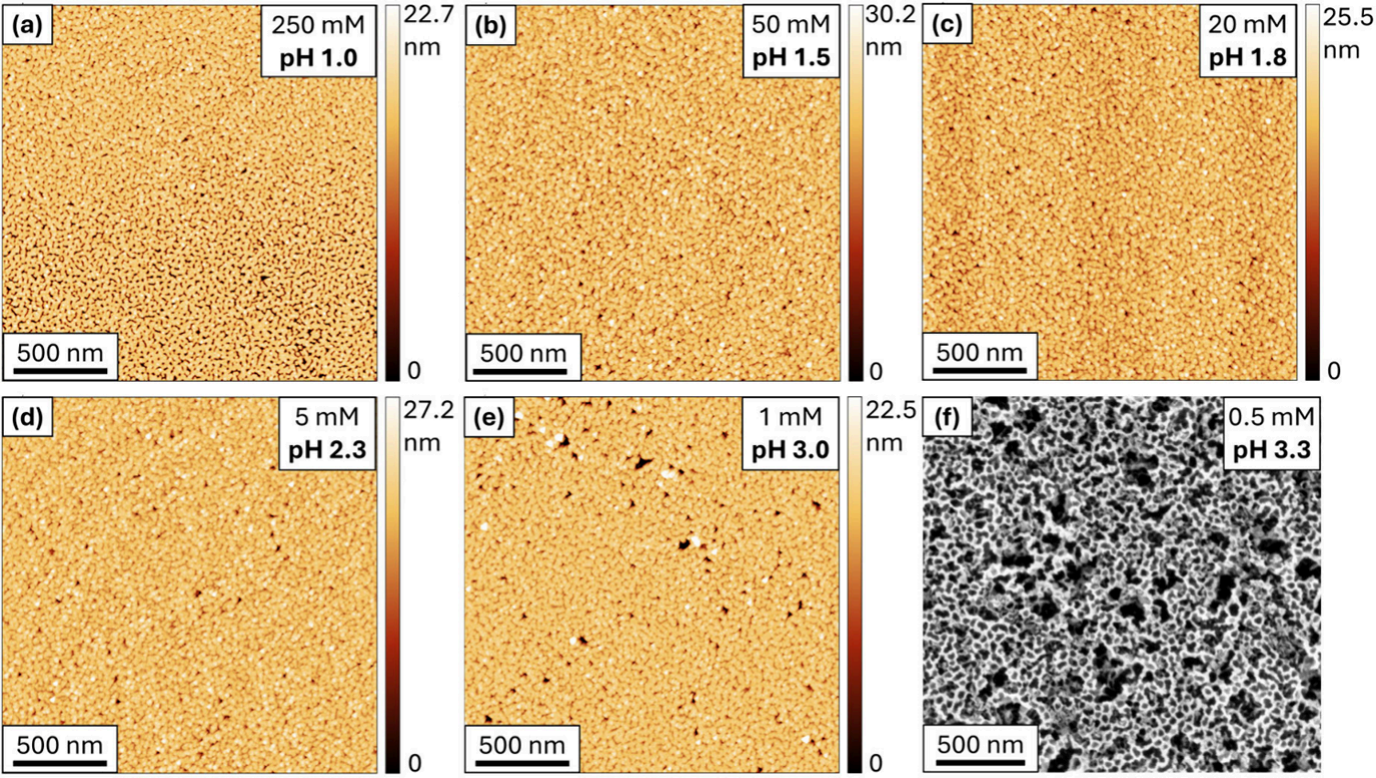
AFM images for 1 μm n-type GaN ([Si] = 1.5 × 10^19^ cm^–3^) etched at 8 V vs CE (2 electrode electrochemical cell) in a range of concentrations of H_2_C_2_O_4_: (a) 250 mM (pH 1), (b) 50 mM (pH 1.5), (c) 20 mM (pH 1.8), (d) 5 mM (pH 2.34), (e) 1 mM (pH 3). (f) SE plan-view image using 0.5 mM (pH 3.3) H_2_C_2_O_4_.

To confirm that this ECE behavior is not due to the increasing solution resistance (*R*_sol_) from decreasing electrolyte concentration (Table S1), equivalent ECE of n-type GaN in 10, 5, and 1 mM solutions of Na_2_C_2_O_4_ was performed (Figure S5). The resulting bulk and surface pore morphologies for these experiments are very similar to that when using 100 mM Na_2_C_2_O_4_, and *R*_sol_ are measured to be similar magnitudes compared to the equivalent concentrations for H_2_C_2_O_4_. The difference observed for H_2_C_2_O_4_ must therefore be due to a chemical/mechanistic change, as otherwise *R*_sol_ would also significantly affect the Na_2_C_2_O_4_ ECE. These findings indicate that the pore structure differences observed in the buffer solutions are not driven by pH, but rather by the relative composition of H_2_C_2_O_4_ and Na_2_C_2_O_4_. Therefore, the difference between ECE performed in H_2_C_2_O_4_ and Na_2_C_2_O_4_ cannot be attributed to the significant differences in pH but must result from the differences in anionic species present in solution due to their differing dissociation in DI water.

### 3.3 Anionic Species

H_2_C_2_O_4_ is often assumed to dissociate into C_2_O_4_^2–^ anions in DI water, implying that the ECE mechanism involves only C_2_O_4_^2–^.^21,22^ While this is true for Na_2_C_2_O_4_, which dissociates completely (eq 2), H_2_C_2_O_4_ is a diprotic weak acid and will therefore partially dissociate in two steps, with each step forming a dynamic equilibrium between products and reactants (eq 3, eq 4). As a result, H_2_C_2_O_4_ yields both HC_2_O_4_^–^ and C_2_O_4_^2–^ in solution, while Na_2_C_2_O_4_ yields only C_2_O_4_^2–^ (ignoring the subsequent hydrolysis of the C_2_O_4_^2–^ anion, as discussed in Section 3.1). This difference in anion composition within the etchants offers a mechanistic explanation for the significantly different ECE behavior observed (i.e., the variation in porous structure is due to the difference in ECE mechanism between HC_2_O_4_^–^ and C_2_O_4_^2–^ anions).

2 $${\mathrm{Na}}_{2}C_{2}O_{4}\to 2{\mathrm{Na}}^{+}+C_{2}{O_{4}}^{2-}$$

3 $$H_{2}C_{2}O_{4}⇌H^{+}+{\mathrm{HC}}_{2}{O_{4}}^{-}$$

4 $${\mathrm{HC}}_{2}{O_{4}}^{-}⇌H^{+}+C_{2}{O_{4}}^{2-}$$

The extent of dissociation in each step for H_2_C_2_O_4_ can be estimated using the acid dissociation constants (*K*_a1_ and *K*_a2_) for each dynamic equilibrium, which relate the concentrations of products and reactants (eq 5, eq 6). Using reported values of *K*_a1_ and *K*_a2_ as 0.056 and 0.00015 M, respectively,^32^ the concentrations of HC_2_O_4_^–^ and C_2_O_4_^2–^ can be calculated for a given initial concentration of H_2_C_2_O_4_ (Table 2).

5 $$K_{a1}=\frac{\left[H^{+}\right]\left[{\mathrm{HC}}_{2}{O_{4}}^{-}\right]}{\left[H_{2}C_{2}O_{4}\right]}$$

6 $$K_{a2}=\frac{\left[H^{+}\right]\left[C_{2}{O_{4}}^{2-}\right]}{\left[{\mathrm{HC}}_{2}{O_{4}}^{-}\right]}$$

**Table 2:** Solution pH for a Given Range of Initial Oxalic Acid Concentrations ([H_2_C_2_O_4_]_o_) and the Effect on the Concentrations of the Two Anionic Dissociation Products and Their Relative Ratio ([HC_2_O_4_^–^]/[C_2_O_4_^2–^])

[H_2_C_2_O_4_]_o_/mM	pH	[H_2_C_2_O_4_]/mM	[HC_2_O_4_^–^]/mM	[C_2_O_4_^2–^]/mM	Anion Ratio
250	1.0	156	93.4	0.150	625
100	1.3	48.1	51.8	0.149	347
50	1.5	18.1	31.7	0.149	213
20	1.8	4.37	15.5	0.147	105
5	2.3	0.381	4.48	0.141	31.7
1	3.0	0.0172	0.865	0.118	7.34
0.5	3.3	0.00439	0.396	0.0997	3.97
0.1	4.0	0.000178	0.0499	0.0499	1.00

The calculated composition data in Table 2 support the hypothesis that ECE using H_2_C_2_O_4_ (at >1 mM concentration) is dominated by HC_2_O_4_^–^ anions (e.g., for 100 mM H_2_C_2_O_4_ there is a factor of 347 more HC_2_O_4_^–^ anions compared to C_2_O_4_^2–^). This is strong evidence of the differing ECE behavior of H_2_C_2_O_4_ and Na_2_C_2_O_4_. As the concentration of H_2_C_2_O_4_ is decreased, the relative abundance of C_2_O_4_^2–^ remains negligible, and the surface structure remains characteristic of HC_2_O_4_^–^ etching. However, at concentrations of 1 mM or below, the proportion of C_2_O_4_^2–^ becomes significant, corresponding to the observed structural shift toward the rougher, more chaotic etching characteristic of C_2_O_4_^2–^-driven ECE (i.e., Na_2_C_2_O_4_). Additionally, dual peaks can be seen in chronoamperometry traces when conducting ECE in <1 mM concentrations of H_2_C_2_O_4_ (Figure S6), signifying the transition from HC_2_O_4_^–^ dominance to C_2_O_4_^2–^. This is further supported by the *Q*_GA_ traces in Figure 4a, where the final *Q*_GA_ values after ECE completion remain relatively constant for pH 1.29, 1.5, and 1.75, until a threshold C_2_O_4_^2–^ relative abundance is reached (somewhere between pH 1.75 and pH 2), at which point the faster etching HC_2_O_4_^–^ is no longer in high enough proportion to dominate the etch, shown by the significantly higher *Q*_GA_ for pH 2 than at all lower pH values.

As discussed previously, the pH of the H_2_C_2_O_4_ solution decreases during ECE, and therefore the concentration of H^+^ increases. According to Le Chatelier’s principle and the dissociation equilibria in eq 3 and eq 4, this increasing concentration of H^+^ shifts both equilibria to the left, reducing the concentration of C_2_O_4_^2–^ and increasing the HC_2_O_4_^–^/C_2_O_4_^2–^ ratio. Indeed, if the H_2_C_2_O_4_ concentration series from Figure 5 is repeated using serial dilutions (where the 250 mM solution is freshly prepared and each subsequent lower concentration solution is diluted from the previously used one), then the onset of C_2_O_4_^2–^ contribution toward ECE at 1 mM is no longer observed from the GaN surface morphologies (Figure S7), consistent with a decrease in pH (increase in concentration of H^+^) and thus an increase in the [HC_2_O_4_^–^]/[C_2_O_4_^2–^] ratio compared to that predicted in Table 2.

To further validate that the transition in porous morphology arises from the changing [HC_2_O_4_^–^]/[C_2_O_4_^2–^] ratio, an etchant solution was prepared by neutralizing 1.5 mmol of H_2_C_2_O_4_ (15 mL of 100 mM) with 3 mmol of NaOH (30 mL of 100 mM), thereby removing all free H^+^ and driving both equilibria (eq 3 and eq 4) fully to the right. Under these conditions, C_2_O_4_^2–^ is the only anionic species in the solution. As expected, the resulting porous morphology is like that produced using Na_2_C_2_O_4_ (Figure S8a–c), and the chronoamperometry trace contains the characteristic dual-peak shape, confirming the role of C_2_O_4_^2–^ as the species responsible for the characteristic, highly irregular porous structure. In addition, ECE was performed in a similar mixed etchant solution of 2.5 mmol of H_2_C_2_O_4_ (25 mL of 100 mM) with 2.5 mmol of NaOH (25 mL of 100 mM) to produce 50 mM NaHC_2_O_4_, therefore possessing a similar [HC_2_O_4_^–^]/[C_2_O_4_^2–^] ratio to the 100 mM solution of H_2_C_2_O_4_ (334 and 347, respectively). As expected based on anion speciation, the pore morphology resulting from this mixed etchant appears the same as that for 100 mM H_2_C_2_O_4_, and the etching current also displays oscillatory behavior (Figure S8d–f).

### 3.4 Extending to Other Etchant Solutions

The proposed mechanism of anion-specific ECE should be valid across a range of etchant species and not solely for H_2_C_2_O_4_ and Na_2_C_2_O_4_. Any acid that dissociates in DI water to produce a single anion species should result in the same ECE behavior and pore morphology as its corresponding salt, which fully dissociates to yield the same anion. This principle applies to any monoprotic acid, as even if it is weak and therefore forms a dynamic equilibrium between the chemical and the anion, only the anion participates directly in the ECE process. One exception to this is if the acid dissociates very poorly, and hence the concentration of anion from the acid is substantially lower than that from the conjugate salt and enters an anion-limited regime, as seen previously for <0.5 mM H_2_C_2_O_4_ (Figure S4). In contrast, poly­(protic acid)­s (e.g., H_2_C_2_O_4_) yield multiple anionic species, with the singly charged anion typically at a dominant abundance. Since the corresponding salt dissociates fully to produce only the highest charged anion, the resulting ECE mechanism is expected to differ from that of the acid. Table 3 summarizes a range of example acid/salt pairs and the expected consistency or divergence in ECE behavior and resulting porous morphology.

**Table 3:** Acid Etchants (H_2_C_2_O_4_, H_2_SO_4_, H_3_PO_4_, HCl, HNO_3_, and CH_3_COOH) and Their Anionic Dissociation Products in DI Water^a^

Acid Etchant	Dissociation Products	Corresponding Salt	Salt Anionic Product	Same Etching Mechanism?
H_2_C_2_O_4_	**HC**_**2**_ **O**_**4**_^**–**^, C_2_O_4_^2–^	Na_2_C_2_O_4_	C_2_O_4_^2–^	No
H_2_SO_4_	**HSO**_**4**_^**–**^, SO_4_^2–^	Na_2_SO_4_	SO_4_^2–^	No
H_3_PO_4_	**H**_**2**_ **PO**_**4**_^**–**^, HPO_4_^2–^, PO_4_^3–^	Na_3_PO_4_	PO_4_^3–^	No
HCl	**Cl** ^**–**^	NaCl	Cl^–^	Yes
HNO_3_	**NO**_**3**_^**–**^	NaNO_3_	NO_3_^–^	Yes
CH_3_COOH	**CH**_**3**_ **COO** ^**–**^	CH_3_COONa	CH_3_COO^–^	Yes

a The dominant (highest concentration) anion is in bold. The corresponding salts are listed along with their anionic dissociation product. An acid and its corresponding salt are predicted to etch via the same mechanism only if the dominant anionic products are the same.

For example, H_2_SO_4_ is a strong acid and so fully dissociates in its first dissociation step (eq 7), but the second step is still governed by a dynamic equilibrium (eq 8) with a dissociation constant of *K*_a2_ ≈ 0.01 M.^32^ Using an approach analogous to that applied for H_2_C_2_O_4_ (Table 2), the calculated ratio of [HSO_4_^–^]/[SO_4_^2–^] for 100 mM H_2_SO_4_ is ∼10.8; hence the majority species is still the singly charged anion, HSO_4_^–^. The ECE mechanism in H_2_SO_4_ is therefore expected to differ from that in Na_2_SO_4_, where SO_4_^2–^ is the sole anion. This is confirmed by SEM images showing distinct differences in pore morphology between n-type GaN etched in H_2_SO_4_ and Na_2_SO_4_ under identical conditions (Figure 6a iii and iv, respectively). This is further supported by the significant observed differences between pore morphologies produced using H_3_PO_4_ and Na_3_PO_4_ etchants (Figure 6a v, vi, respectively).

7 $$H_{2}{\mathrm{SO}}_{4}\to H^{+}+{{\mathrm{HSO}}_{4}}^{-}$$

8 $${{\mathrm{HSO}}_{4}}^{-}⇌H^{+}+{{\mathrm{SO}}_{4}}^{2-}$$

**Figure 6:**
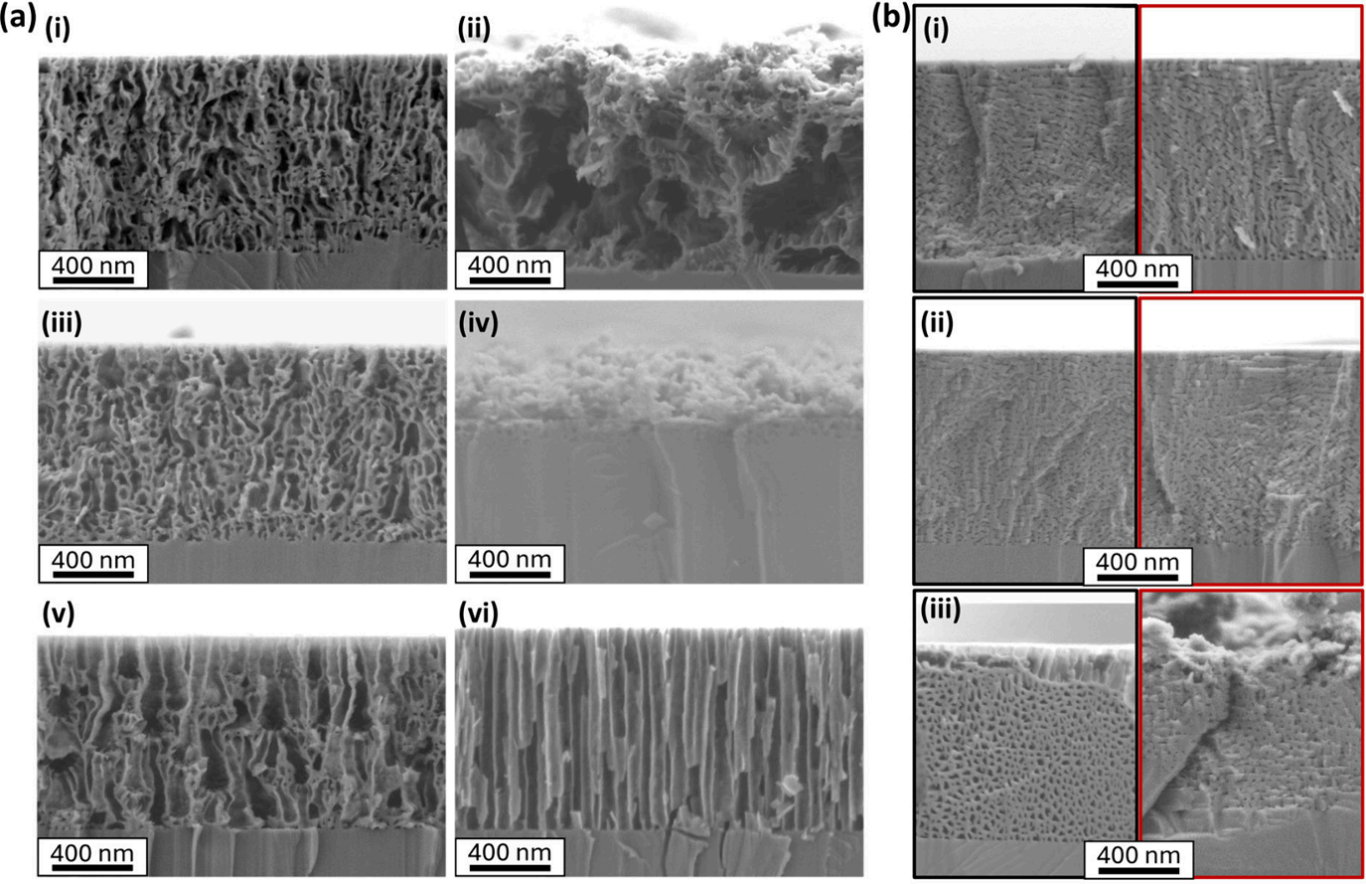
Cross-section SEM images for 1 μm n-type GaN ([Si] = 1.5 × 10^19^ cm^–3^) etched at 8 V vs Ag/AgCl in 100 mM (a) acids that dissociate to >1 anion in equilibria and their conjugate bases: (i) H_2_C_2_O_4_, (ii) Na_2_C_2_O_4_, (iii) H_2_SO_4_, (iv) Na_2_SO_4_, (v) H_3_PO_4_, (vi) Na_3_PO_4_; (b) acids that dissociate to a single anion and their conjugate bases: (i) (black) HNO_3_, (red) KNO_3_, (ii) (black) HCl, (red) NaCl, (iii) (black) CH_3_COOH, (red) CH_3_COONa.

In contrast, HCl is a strong, monoprotic acid and therefore dissociates fully to yield Cl^–^, the same anion (and at the same concentration) as that provided by NaCl. As predicted by the anion-specific ECE mechanism, HCl and NaCl result in indistinguishable porous structures (Figure 6b ii, Figure S9), both forming a lateral pore morphology. This is also true for KCl, which was compared to NaCl in Figure 3 to reveal identical pore morphologies despite the different cation species. A similar observation applies to HNO_3_ and NaNO_3_, both of which yield nearly identical morphologies (Figure 6b i, Figure S9), consistent with NO_3_^–^ being the only active anion toward ECE in both etchants. The behavior of NO_3_^–^ and Cl^–^ anions appears to be identical, suggesting that both anions operate within the same ECE mechanism.

CH_3_COOH is a weak, monoprotic acid that dissociates to produce a single anionic species (CH_3_COO^–^), existing in dynamic equilibrium with its undissociated form. Compared to CH_3_COONa, its conjugate salt, both cross sectional images show a similar, laterally etched morphology (Figure 6b iii), indicating a comparable ECE mechanism. However, CH_3_COOH results in significantly wider pore diameters. This is a similar observation to that discussed for KOH and NaOH (Figure 3), where overall morphology is consistent but there are differences in pore size. The GaN surface structure produced by CH_3_COOH appears similar to that of H_2_C_2_O_4_- and H_3_PO_4_-driven ECE, though with slightly greater apparent roughness (Figure S9). In contrast, CH_3_COONa yields an extremely roughened, irregular-appearing surface. Despite both etchants involving only the CH_3_COO^–^ anion, these structural differences can be explained by the low dissociation constant of CH_3_COOH (*K*_a_ = 1.75 × 10^–5^ M).^32^ This means that a 100 mM solution of CH_3_COOH generates only ∼1.3 mM CH_3_COO^–^, compared to 100 mM in the fully dissociated CH_3_COONa solution. This substantial difference in active anion concentration accounts for the differences in surface structure and pore diameter within the same overall ECE mechanistic regime. Indeed, when ECE is instead performed in 1.3 mM CH_3_COONa, the bulk pore structure then appears very similar to that of 100 mM CH_3_COOH (Figure S10). Surface differences remain for 1.3 mM CH_3_COONa compared to 100 mM CH_3_COOH, but these are less pronounced and likely arise from the variation in solution composition (e.g., conductivity, mass transport, and presence of undissociated CH_3_COOH molecules). Further, the surface behavior described when using Na_2_C_2_O_4_ (Figure S1) and the minor surface pore size variations resulting from NaOH and KOH etchants (Figure 3c iii, d iii) suggest that the n-type GaN surface may be particularly sensitive to minor changes in etchant solution concentration and composition.

A further insight into anion-specific behavior is the presence of *I*_GA_ oscillations in the chronoamperometric ECE data. Previously attributed to the current-burst model, and acting as its most significant evidence in n-type GaN ECE,^12,16,17,19^ these oscillations appear instead to correlate with the presence of dynamic equilibria between multiple active anions. Figure 7 shows *I*_GA_ traces for the series of etchant solutions discussed from Figure 6. Oscillations are observed exclusively in etchants containing at least one dynamic equilibrium between two active anions, specifically H_2_C_2_O_4_, H_2_SO_4_, and H_3_PO_4_ (Figure 7a i, iii, v). H_2_C_2_O_4_ and H_2_SO_4_ each form a single dissociation equilibrium between two anions and display relatively uniform oscillation patterns, although the third peak for H_2_SO_4_ exhibits a nonrandom, sudden onset of irregular, high-frequency fluctuations, suggesting a transient but significant event, followed by significantly lower amplitude oscillations. However, H_3_PO_4_, with two equilibria and three anionic species, exhibits more complex, nonuniform oscillations. Conversely, etchants that dissociate fully into a single anion, or only form one equilibrium between an anion and its undissociated (ECE inactive) acid, show no oscillatory behavior (Figure 7a ii, iv, vi, Figure 7b).

**Figure 7:**
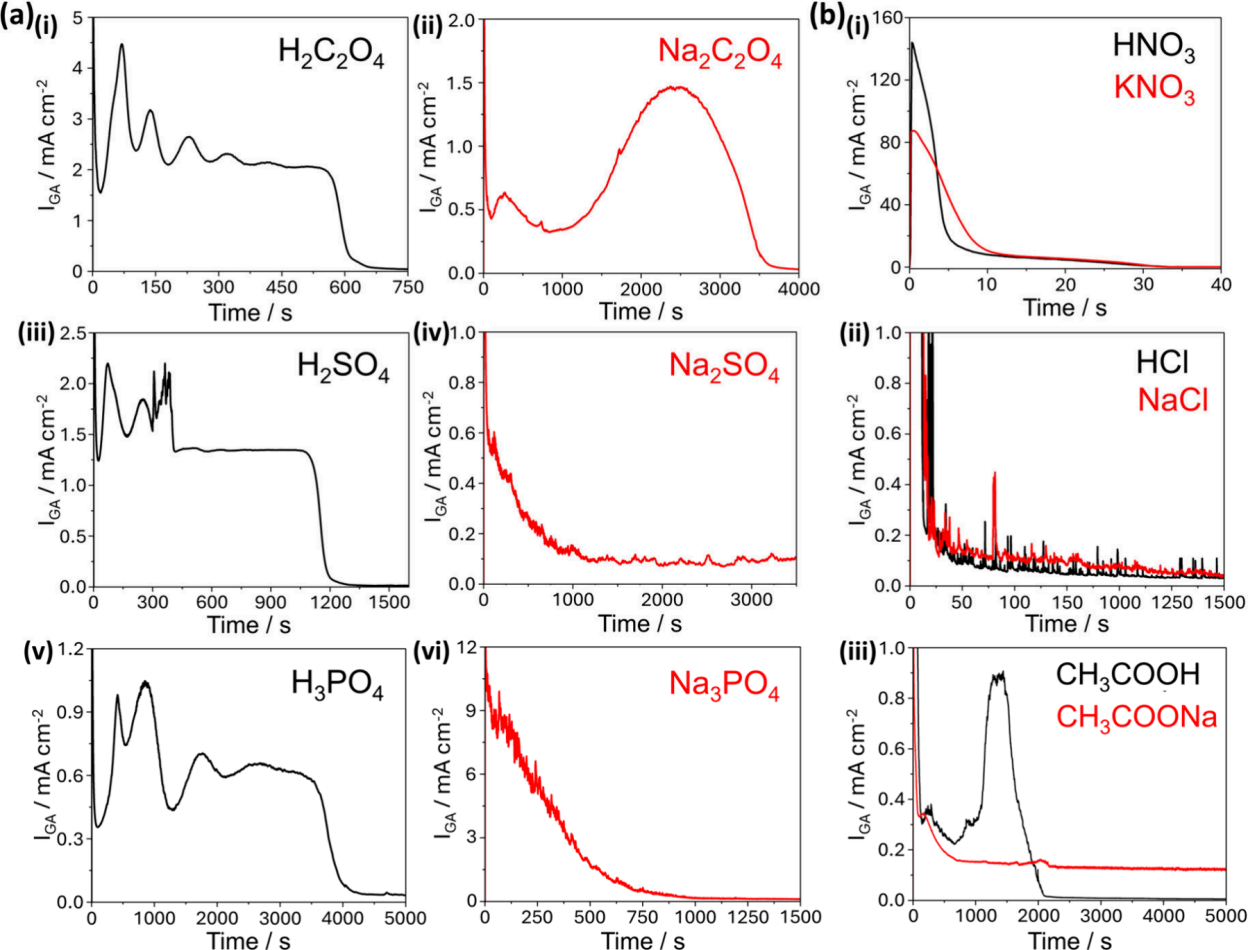
Chronoamperometry data for ECE of 1 μm n-type GaN ([Si] = 1.5 × 10^19^ cm^–3^) etched at 8 V vs Ag/AgCl in 100 mM (a) acids which dissociate to >1 anion in equilibria and their conjugate bases: (i) H_2_C_2_O_4_, (ii) Na_2_C_2_O_4_, (iii) H_2_SO_4_, (iv) Na_2_SO_4_, (v) H_3_PO_4_, (vi) Na_3_PO_4_; (b) acids that dissociate to a single anion and their conjugate bases: (i) (black) HNO_3_, (red) KNO_3_, (ii) (black) HCl, (red) NaCl, (iii) (black) CH_3_COOH, (red) CH_3_COONa.

These findings provide speculative evidence that the ECE current oscillations originate from the presence of multiple ECE-active anions rather than from the current-burst model. In this case, the current-burst model is unlikely to be an accurate representation of how an n-type GaN ECE occurs. However, like the current-burst model, this new explanation would still require local changes that occur in phase with one another across an entire sample to produce such oscillations, suggesting that the origin and ECE mechanism are likely more complex and still not entirely understood. Indeed, this does not rule out alternative origins of the oscillations, such as oxide formation or gas evolution feedback, other than from the notion that these two possibilities would be expected to occur for all etchants, rather than for specifically the three polyprotic acids out of 12 chemicals addressed in this work. It is reasonable to suggest that the true mechanism may involve a combination of many of these interrelated processes and is therefore an extension of the current-burst model, reflecting the increased complexity of n-type GaN ECE (which operates across a broad range of etchants) compared to Si ECE (which proceeds exclusively in HF etchant).^17,19^ However, all mechanistic evidence reported for n-type GaN ECE, including this study and the current-burst model, relies on correlative data; hence, this is an area that still requires significant further research.

A speculative overall trend can be proposed linking the porous morphology to the ligating strength of the etchant anion toward Ga^3+^ complexation. During ECE, Ga–N bonds are broken, forming aqueous Ga^3+^ (eq 1), which may then oxidize or hydrolyze with water-derived species or form complexes with etchant anions. The rate and proportion of Ga complexation with etchant is therefore expected to influence the ECE dynamics and resulting porous morphology. Given that Ga^3+^ is a hard Lewis acid, strong binding ligands are expected to be hard bases, as well as those which chelate and possess localized donor electrons.^33,34^ For example, C_2_O_4_^2–^ fits these criteria well. Interestingly, the C_2_O_4_^2–^ etchant produces highly porous structures with no obvious directional preference (Figure 6a ii), consistent with rapid Ga^3+^ complexation across all surfaces, leaving insufficient time for competing reactions or facet-specific passivation to hinder the etch. In contrast, HC_2_O_4_^–^ has only moderate ligating strength and results in a morphology with reduced porosity and increased directional preference (Figure 6a i). Weakly coordinating anions such as Cl^–^ and NO_3_^–^ then yield low porosity morphologies with a consistent repeating pattern, clearly dominated by lateral pore features (Figure 6b i, ii). While this correlation follows expected chemical trends and explains the significant difference in ECE behavior between different etchant chemicals (which the current-burst theory does not), it presently remains speculative and will require further mechanistic investigation.

### 3.5 Broader Implications

A deeper understanding of ECE of n-type GaN is essential for optimizing process parameters, enabling the design of new porous morphologies, and expanding the range of potential applications. The differences in ECE behavior for a range of anions, even between closely related anions such as HC_2_O_4_^–^ and C_2_O_4_^2–^, highlight the importance of the active etchant species on the underlying mechanism and resulting porous morphology. In this work, it is demonstrated that ECE in H_2_C_2_O_4_ at typical concentrations (∼100–250 mM) proceeds via the HC_2_O_4_^–^ anion, rather than C_2_O_4_^2–^, hence enabling a more accurate correlation between etchant identity and ECE behavior. While all previous studies using H_2_C_2_O_4_ have reasonably assumed C_2_O_4_^2–^ to be the only anion species taking part in the ECE mechanism,^21,22^ this new evidence suggests that this overlooks the influence of incomplete acid dissociation, and therefore will lead to misinterpretation of chelation and complexation processes.

The observed ECE current oscillations can be correlated with dissociation equilibria between two or more anionic species; therefore, the oscillatory peaks and troughs could be driven by local dynamic shifts of the equilibria, and hence the relative concentrations of anions at the n-type GaN surface. If this is the case, the oscillations directly reflect transitions between different ECE mechanisms (i.e., in H_2_C_2_O_4_, the peaks may correspond to a high [HC_2_O_4_^–^]/[C_2_O_4_^2–^] ratio, while the troughs correspond to a lower ratio). While this currently remains speculative based on correlation, it would mean that larger amplitude and/or different frequency oscillations could notably influence the resulting porous structure, posing a challenge for reproducible etching of large n-type GaN wafers such as in commercial and scale-up applications. However, this limitation can be mitigated by employing flow-based electrochemical systems in which the etchant is continuously circulated over the sample to maintain uniform composition and suppress local fluctuations. Alternatively, ECE current oscillations could be simply due to the etchant containing more than one active anion with different and competing ECE mechanisms. ECE was performed in Na_2_C_2_O_4_ mixed with other fully dissociating salts (Na_2_SO_4_ or NaCl), but no oscillations were observed in any chronoamperometry traces of these mixtures (Figure S11).

Since the ECE mechanism has been shown to be solely dependent on the active anion species, hazardous etchants can be substituted for safer, cheaper, and environmentally benign alternatives (e.g., NaCl in place of HCl), broadening the practical utility of ECE for scalable and sustainable porous GaN fabrication.

The ability to engineer bespoke porous architectures with application-specific properties is a critical step toward the development of next-generation high-efficiency devices. The mechanistic insight provided here offers a new understanding of the role of etchant speciation in porous structure formation, providing new opportunities for controlling morphology through chemical design.

## 4 Conclusions

The ECE mechanism for porous GaN formation has been proven to be primarily influenced by the anionic composition of the etchant solution, with cationic species and solution pH displaying a minimal impact. The stepwise dissociation of polyprotic acids (e.g., H_2_C_2_O_4_) yields multiple anionic species, which compete during ECE. While the singly charged anion typically dominates due to its higher abundance and faster etching rate, the porous morphology can be tuned by reducing the etchant concentration, thereby shifting the dissociation equilibria to favor more highly charged anions. Alternatively, mixing a poly­(protic acid) with its fully dissociating corresponding salt, forming a pH buffer solution, enables the controlled tuning of anion concentration ratios without compromising the total etchant concentration. This mechanistic understanding improves the ability to rationally select and compare etchant systems and provides a pathway to tailor porous GaN architectures for specific device applications.

Current oscillations generated by ECE were found exclusively in the presence of dynamic anion equilibria. The complexity of the oscillations also increased with the number of anionic species. No etchant that produces only a single active anion species generated observable current oscillations. These findings speculatively correlate current oscillations with the dynamically shifting concentrations of multiple active etchant anions, challenging the previously inferred origin of surface passivation by Ga_2_O_3_ formation, as proposed by the current-burst model. These new insights establish a predictive framework for understanding and controlling porous GaN etching through anion-specific mechanisms. This refined understanding opens pathways to scalable, reproducible, and chemically tunable ECE processes, with broad applicability across electronic and optoelectronic devices.

## Supplementary Material



## Notes

Chronoamperometry and SEM characterization when using Na_2_C_2_O_4_ etchants at different ECE durations (Figure S1); AFM image after ECE with 100 mM H_2_C_2_O_4_ (Figure S2); AFM and SE images after ECE in oxalate pH buffers with varying set pH (Figure S3); SE images after ECE in H_2_C_2_O_4_ with varying concentrations (Figure S4); SEM and chronoamperometry data for ECE in low concentrations of Na_2_C_2_O_4_ (Figure S5); chronoamperometry and integrated charge data for ECE in H_2_C_2_O_4_ with varying concentrations (Figure S6); AFM images after ECE in H_2_C_2_O_4_ with varying concentrations using serial dilutions of used solution (Figure S7); SE images and chronoamperometry for ECE in varying mixtures of H_2_C_2_O_4_ and NaOH (Figure S8); plan-view SE images for ECE in different etchants (Figure S9); SE images after ECE in 1.3 mM CH_3_COONa (Figure S10); chronoamperometry and cross-section SEM images from ECE with mixed, fully dissociating etchants (Figure S11) (PDF)
