# Supplementary material for: Porous GaN: Anion-Specific Electrochemical Etching Mechanisms and Morphological Control
Source: ACS Appl Mater Interfaces. 2025 Nov 13;17(47):64931–41. doi: 10.1021/acsami.5c18520 (PMC12673525; doi:10.1021/acsami.5c18520)
Supplement: Supplementary file 1 [file am5c18520_si_001.pdf]

Supporting Information for:

Porous GaN: Anion-Specific Electrochemical Etching Mechanisms and Morphological Control

Thom R. Harris-Lee,[a*] Ben Thornley,[a] Jiawei Zhang,[a] Menno J. Kappers[a] and Rachel A. Oliver[a]

[a] Department of Materials Science and Metallurgy, University of Cambridge, Cambridge, CB3 0FS, UK

*trh51@cam.ac.uk

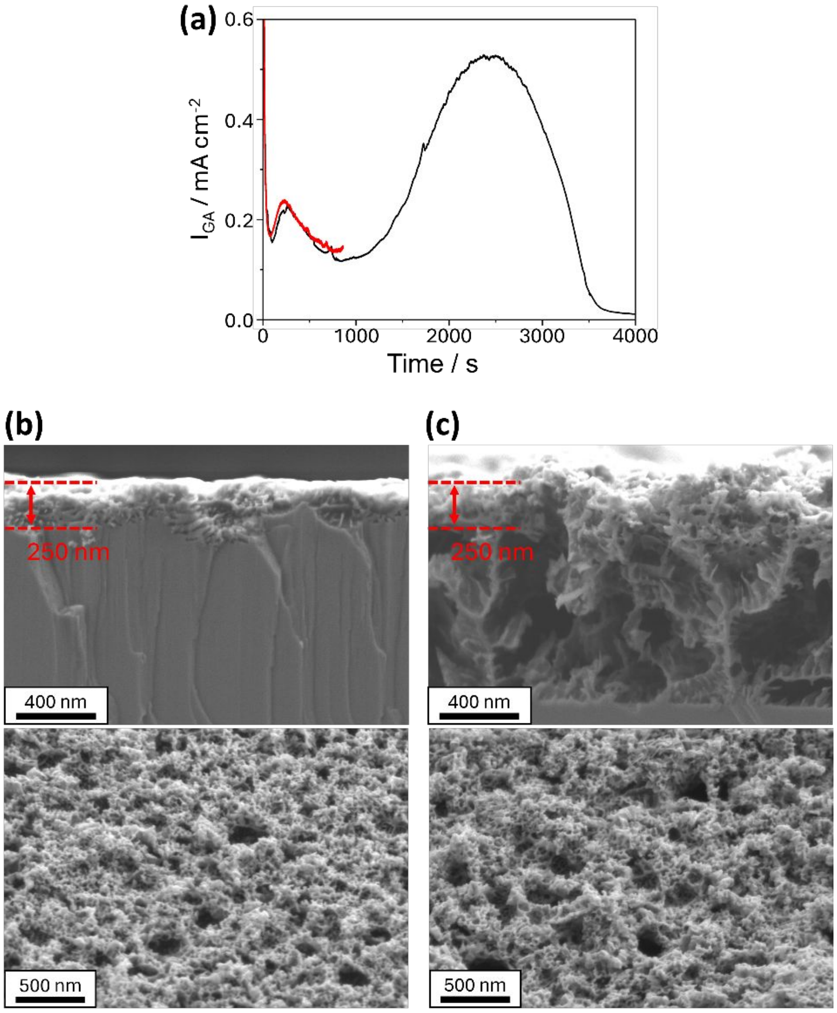

Figure S1. (a) Chronoamperometry data for ECE of 1 µm n-type GaN ([Si] = 1.5x10^{19} cm^{-3}) etched at 8 V vs Ag/AgCl in 0.1 M $Na_2C_2O_4$ until (red) end of the first I_{GA} peak, (black) ECE completion. SEM cross-section and plan view images for the porous GaN morphologies from (b) ECE until end of first I_{GA} peak, (c) ECE completion.

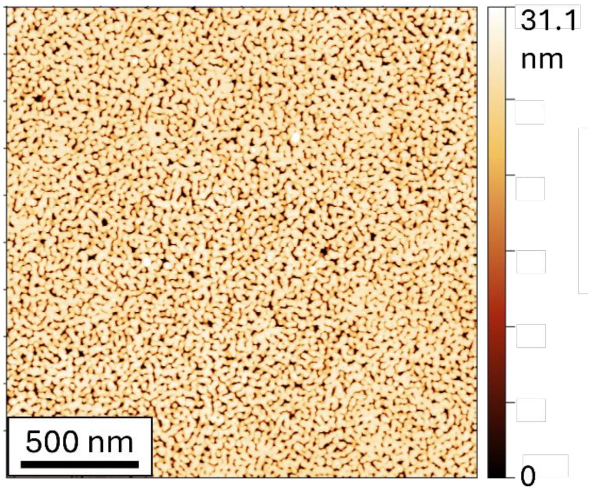

Figure S2. AFM image of porous GaN etched at 8 V vs Ag/AgCl in 0.1 M $H_2C_2O_4$.

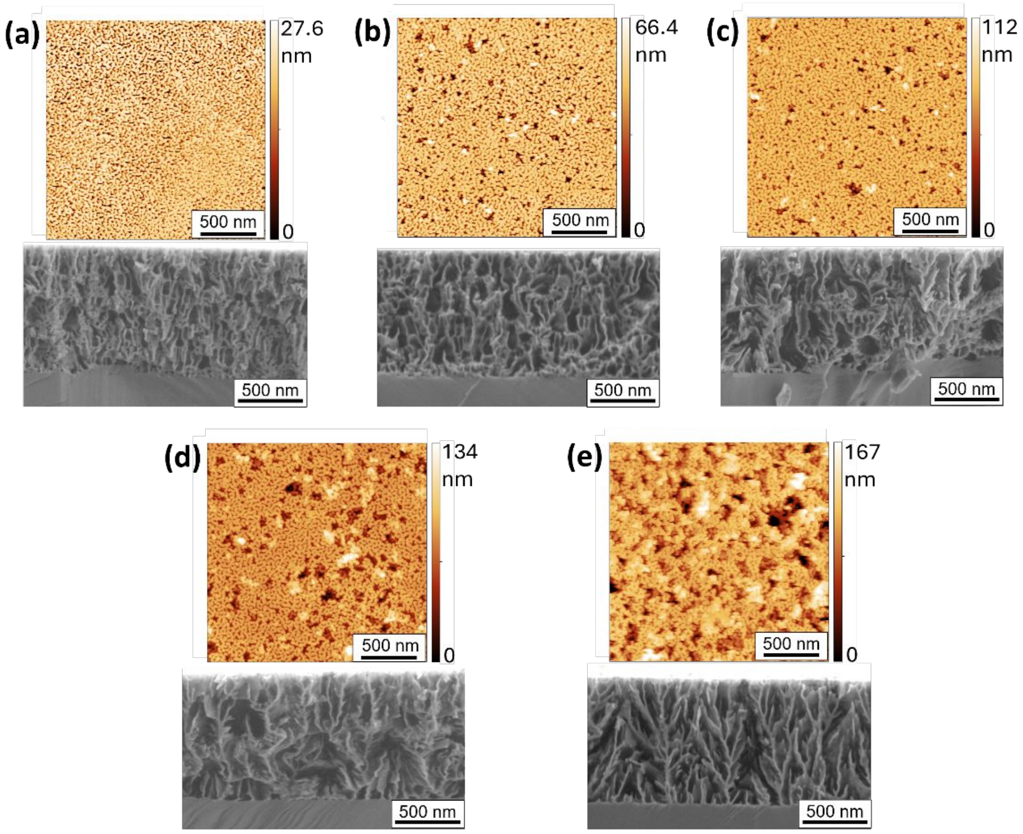

Figure S3. AFM and cross-section SE images for porous GaN samples etched at 8 V vs Ag/AgCl in oxalate buffer solutions with pH values (a) 1, (b) 1.29, (c) 1.5, (d) 1.75, (e) 2.

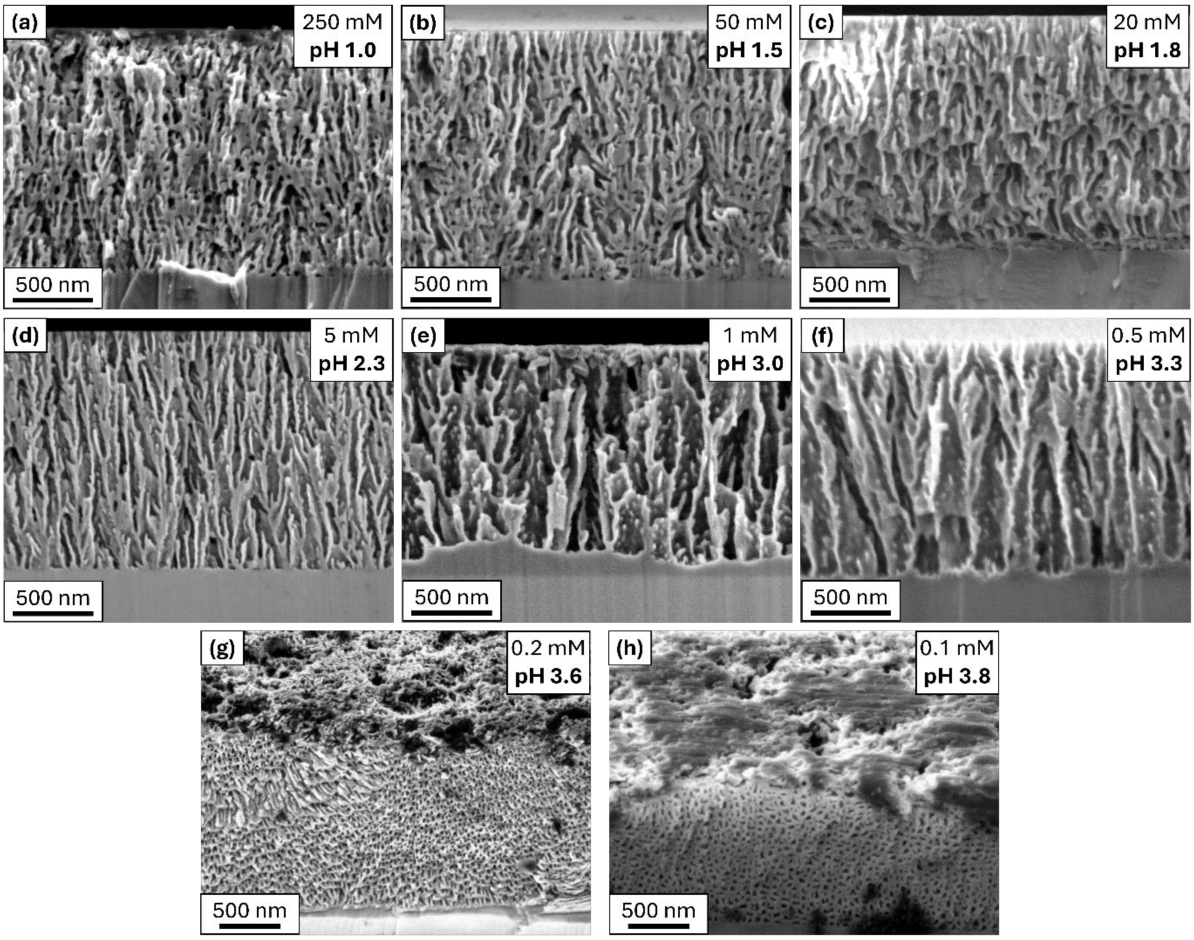

Figure S4. Cross-section SE images for 1 µm n-type GaN ([Si] = 1.5x10^{19} cm^{-3}) etched at 8 V vs CE (2 electrode electrochemical cell) in a range of concentrations of $H_2C_2O_4$: (a) 250 mM (pH 1), (b) 50 mM (pH 1.5), (c) 20 mM (pH 1.8), (d) 5 mM (pH 2.34), (e) 1 mM (pH 3), (f) 0.5 mM (pH 3.3). 45 degree tilted SE image for $H_2C_2O_4$ ultra low concentrations (g) 0.2 mM (pH 3.6), (h) 0.1 mM (pH 3.8).

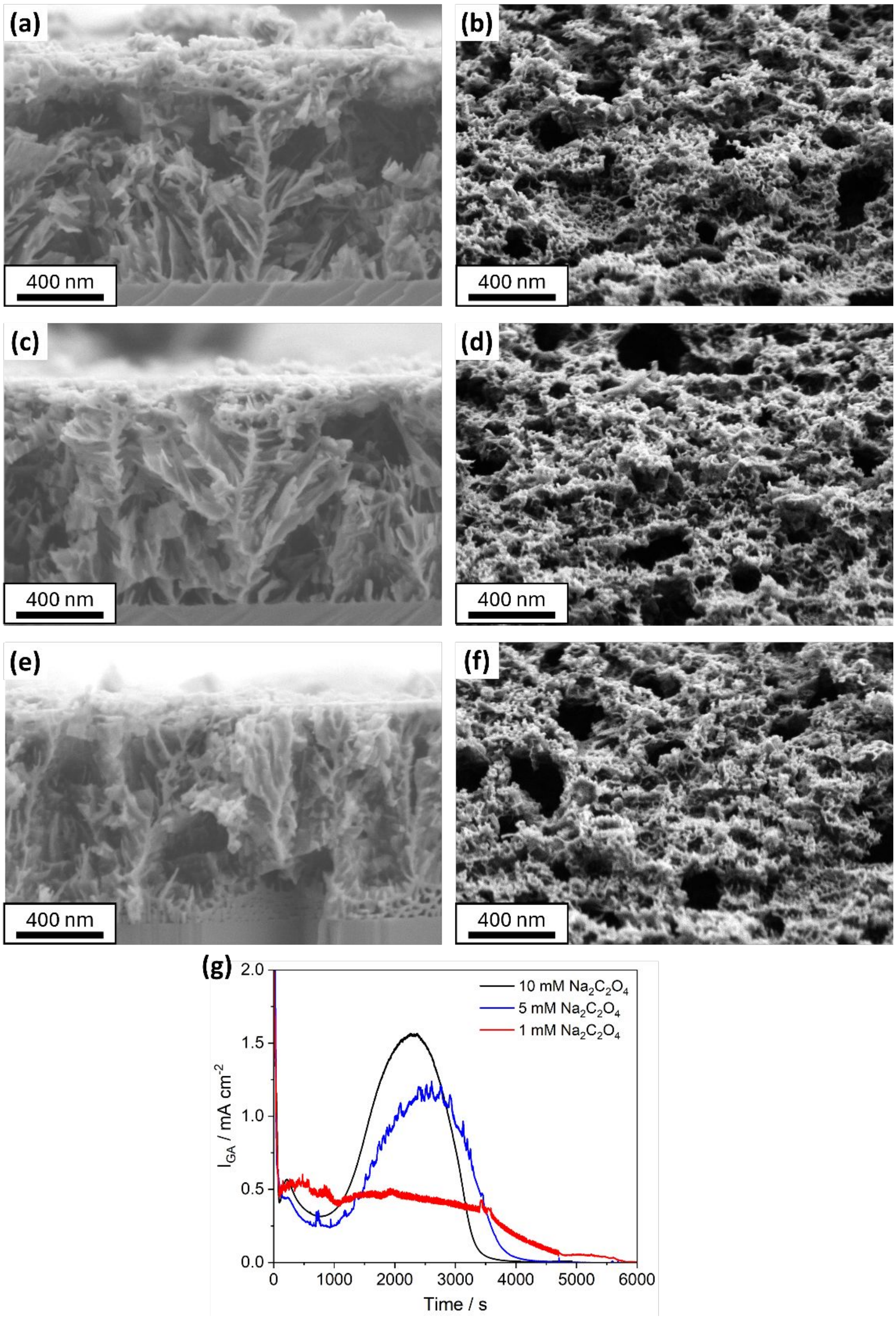

Figure S5. SEM (a,c,e) cross section, (b,d,f) plan view images, (g) ECE chronoamperometry data for 1 µm n-type GaN ([Si] = 1.5x10^{19} cm^{-3}) etched at 8 V vs Ag/AgCl in $Na_2C_2O_4$ solution with concentration (a,b) 10 mM, (c,d) 5 mM, (e,f) 1 mM

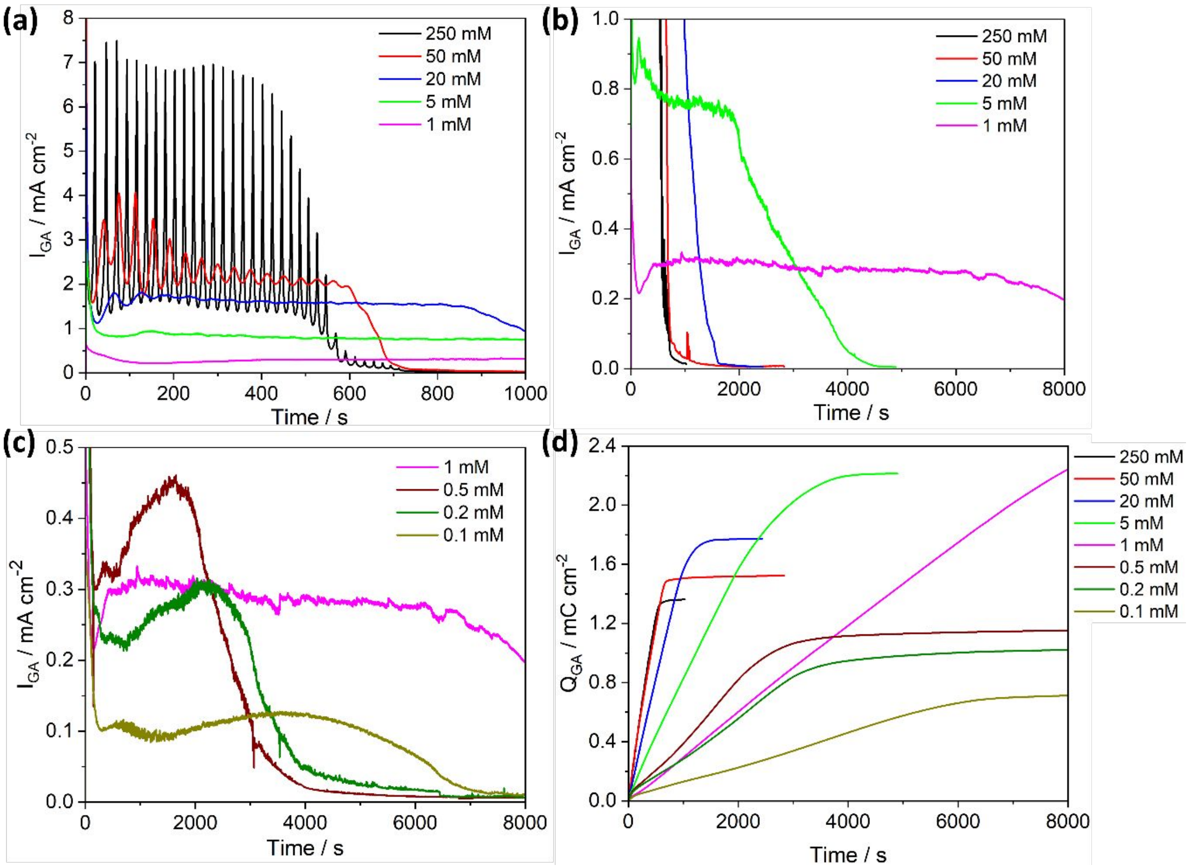

Figure S6. Chronoamperometry data for ECE of 1 µm n-type GaN ([Si] = 1.5x10^{19} cm^{-3}) etched at 8 V vs CE in a range of concentrations of $H_2C_2O_4$: (a) 250 mM – 1 mM for 1000 s duration, (b) 250 mM – 1 mM for 8000 s duration, (c) 1 mM – 0.1 mM. (d) Integrated I_{GA} (Q_{GA}) traces for 250 mM – 0.1 mM concentrations of $H_2C_2O_4$.

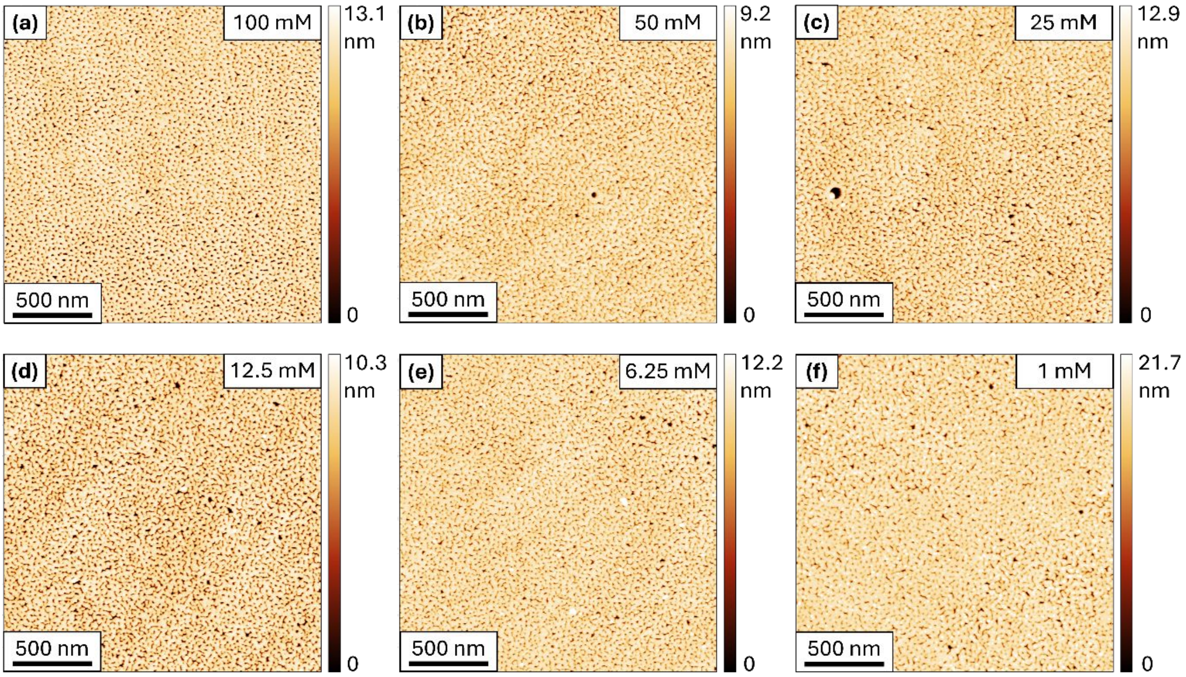

Figure S7. AFM images for 1 µm n-type GaN ([Si] = 1.5×10^{19} cm^{-3}) etched at 8 V vs CE (2 electrode electrochemical cell) in a range of concentrations of $H_2C_2O_4$, with each subsequent solution made by a dilution of the previously used (higher concentration) solution: (a) 100 mM, (b) 50 mM, (c) 25 mM, (d) 12.5 mM, (e) 6.25 mM, (f) 1 mM.

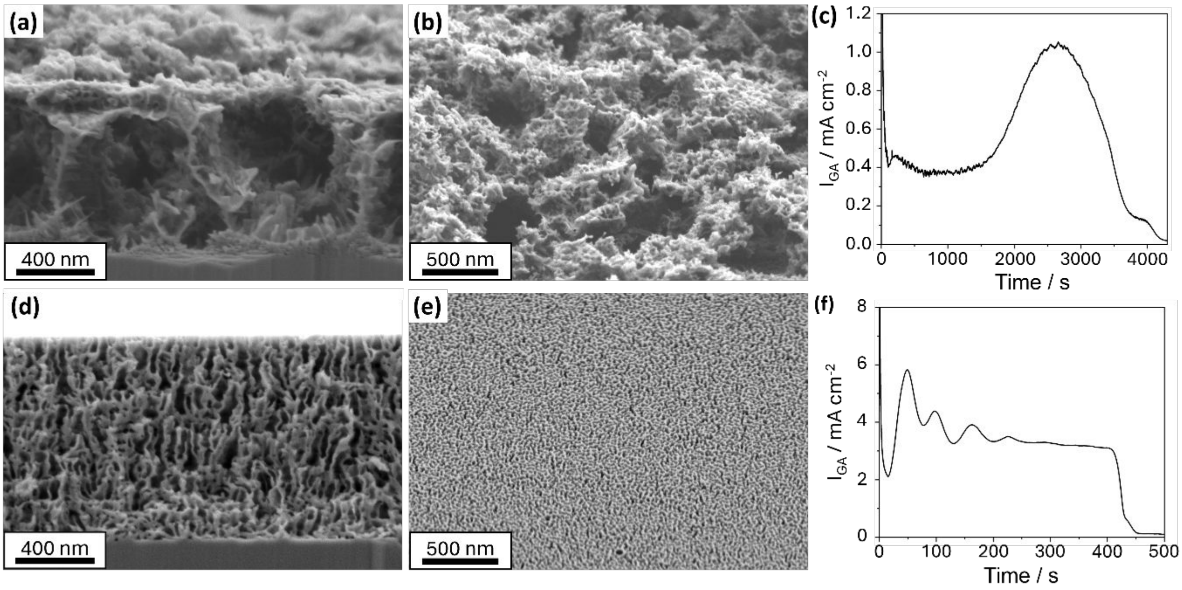

Figure S8. SEM (a,d) cross section, (b,e) plan view images, (c,f) ECE chronoamperometry data for 1 µm n-type GaN ([Si] = 1.5×10^{19} cm^{-3}) etched at 8 V vs Ag/AgCl in an etchant solution consisting of a mixture of (a-c) 33 mM $H_2C_2O_4$ and 66 mM NaOH (to make 33 mM of $Na_2C_2O_4$), (d-f) 50 mM $H_2C_2O_4$ and 50 mM NaOH (to make 50 mM $NaHC_2O_4$).

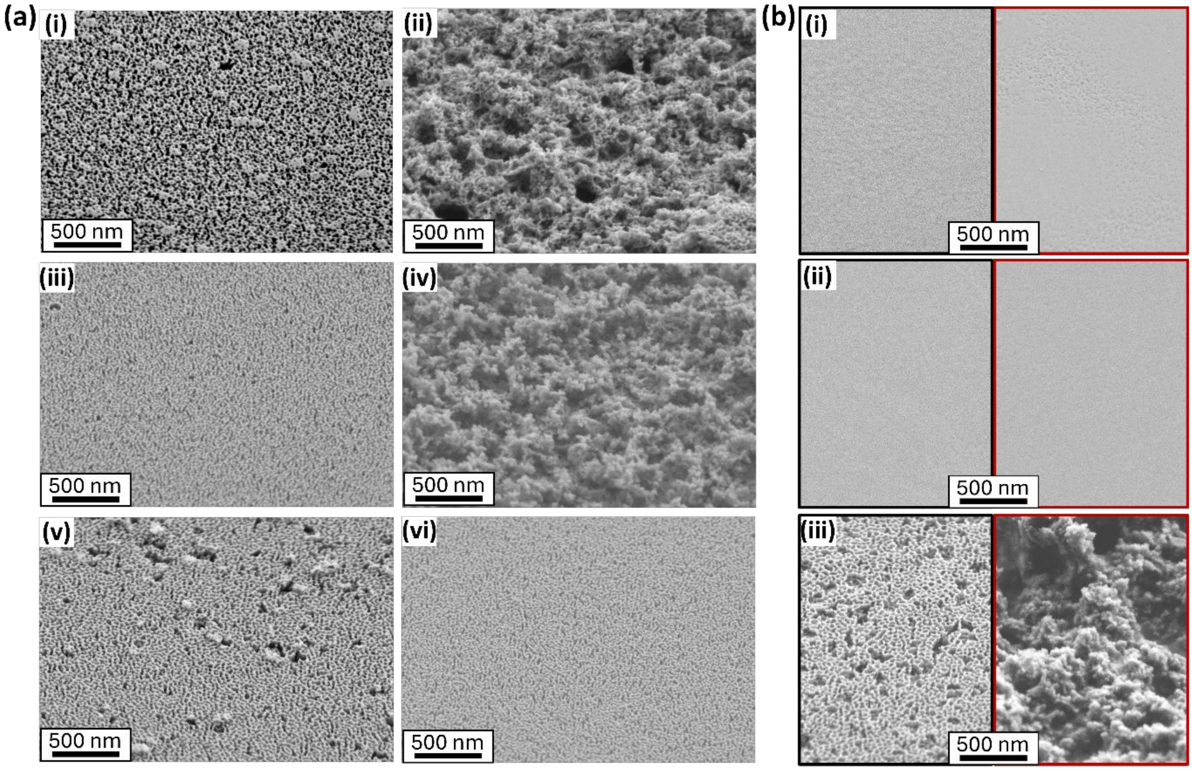

Figure S9. Plan-view (45° tilt) SE images for 1 μm n-type GaN ([Si] = 1.5×10^{19} cm^{-3}) etched at 8 V vs Ag/AgCl in 100 mM (a) Acids which dissociate to >1 anion in equilibria, and their conjugate bases: (i) $H_2C_2O_4$, (ii) $Na_2C_2O_4$, (iii) H_2SO_4, (iv) Na_2SO_4, (v) H_3PO_4, (vi) Na_3PO_4; (b) Acids which dissociate to a single anion, and their conjugate bases: (i) (black) HNO_3, (red) KNO_3, (ii) (black) HCl, (red) NaCl, (iii) (black) CH_3COOH, (red) CH_3COONa.

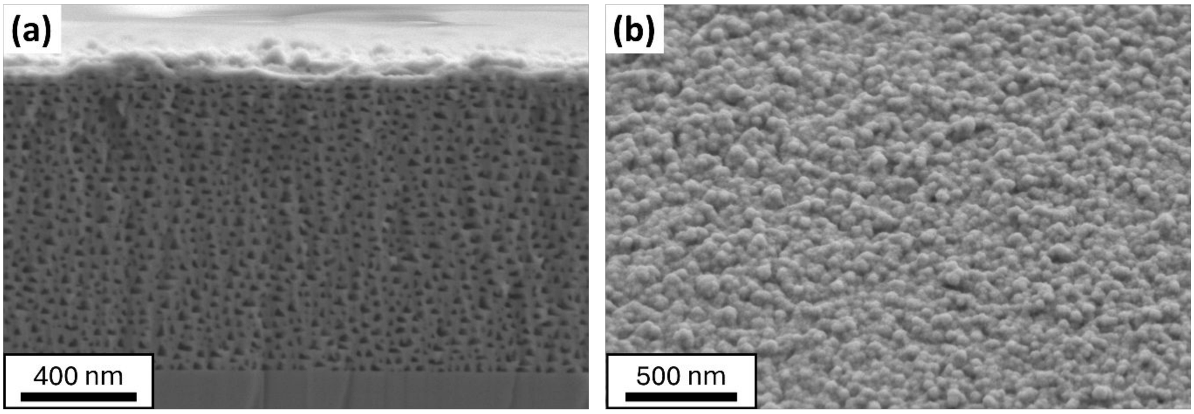

Figure S10. SE (a) cross-section, (b) plan view images of 1 µm n-type GaN ([Si] = 1.5x10^{19} cm^{-3}) etched at 8 V vs Ag/AgCl in 1.3 mM CH_3COONa.

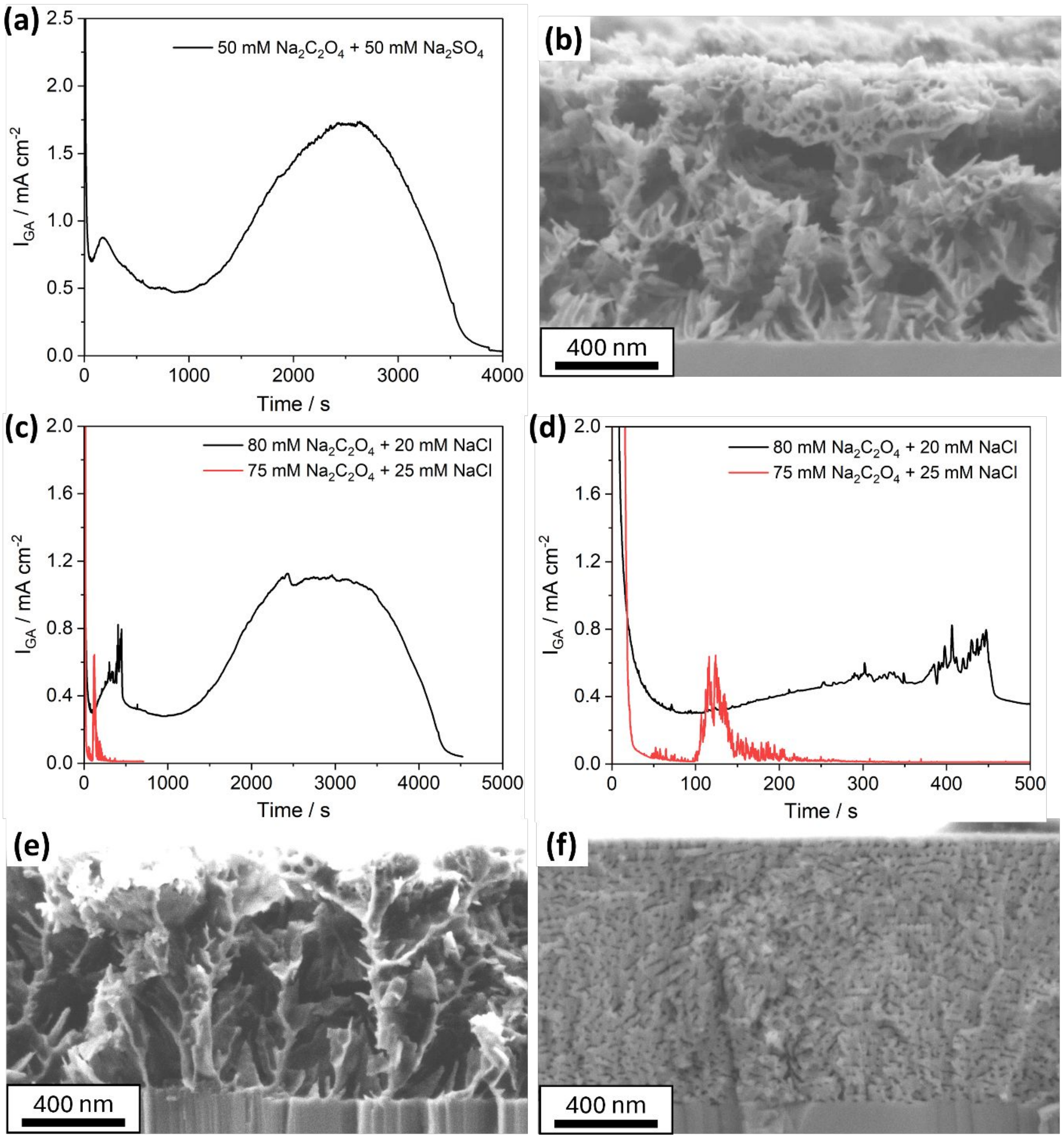

Figure S11. Chronoamperometry (a,c,d) and cross-section SEM images (b, e, f) for 1 µm n-type GaN ([Si] = 1.5×10^{19} cm^{-3}) etched at 8 V vs Ag/AgCl in etchant mixtures of (a,b) 50 mM $Na_2C_2O_4$ + 50 mM H_2SO_4, (c,d) 80 mM $Na_2C_2O_4$ + 20 mM NaCl, and 75 mM $Na_2C_2O_4$ + 25 mM NaCl (showing durations of 4500 s and 500 s), (e) 80 mM $Na_2C_2O_4$ + 20 mM NaCl, (f) 75 mM $Na_2C_2O_4$ + 25 mM NaCl.

Table S1. Solution resistance (R_{sol}), measured by electrochemical impedance spectroscopy, for etchant solutions with varying concentration of $H_2C_2O_4$ or $Na_2C_2O_4$.

Concentration / mM	100	50	20	10	5	1
$H_2C_2O_4$ R_{sol} / Ω	9.89	19.58	52.90	-	222.50	830.82
$Na_2C_2O_4$ R_{sol} / Ω	13.04	-	-	121.12	262.93	1128.04
